# CWH43 Suppresses Ferroptosis-Associated Vulnerability Through Redox Adaptation in Colorectal Cancer

**DOI:** 10.3390/ijms27198830

**Published:** 2026-10-02

**Authors:** Kuei-Yen Tsai, Po-Li Wei, Cheng-Chin Lee, Uyanga Batzorig, Chien-Yu Huang, Yu-Jia Chang

**Affiliations:** 1 Graduate Institute of Clinical Medicine, College of Medicine, Taipei Medical University, Taipei 11031, Taiwan; leiftsai@gmail.com (K.-Y.T.);; 2 Department of Surgery, School of Medicine, College of Medicine, Taipei Medical University, Taipei 11031, Taiwan; poliwei@tmu.edu.tw; 3 Division of General Surgery, Department of Surgery, Shuang Ho Hospital, Taipei Medical University, New Taipei City 235041, Taiwan; 4 Division of Colorectal Surgery, Department of Surgery, Taipei Medical University Hospital, Taipei Medical University, Taipei 11031, Taiwan; 5 Center for Precision Medicine and Translational Cancer Research, Department of Medical Research, Taipei Medical University Hospital, Taipei Medical University, Taipei 11031, Taiwan; 6 Graduate Institute of Cancer Biology and Drug Discovery, Taipei Medical University, Taipei 11031, Taiwan; 7 Taipei Cancer Center, Taipei Medical University, Taipei 11031, Taiwan; 8 Department of Dermatology, Department of Cellular and Molecular Medicine, UCSD Stem Cell Program, University of California San Diego, La Jolla, CA 92093, USA; buyangaaa@yahoo.com; 9 School of Medicine, National Tsing Hua University, Hsinchu 300044, Taiwan; 10 Institute of Molecular and Cellular Biology, National Tsing Hua University, Hsinchu 300044, Taiwan; 11 Department of Pathology, Wan Fang Hospital, Taipei Medical University, Taipei 116079, Taiwan; 12 Cell Physiology and Molecular Image Research Center, Wan Fang Hospital, Taipei Medical University, Taipei 116079, Taiwan

**Keywords:** biomarker, colorectal cancer, chemotherapy, resistance, ferroptosis, NRF2, SLC7A11

## Abstract

Ferroptosis is an iron-dependent form of regulated cell death that has emerged as a potential vulnerability in cancer cells under therapeutic stress. However, the mechanisms by which colorectal cancer cells modulate ferroptosis susceptibility remain incompletely understood. In this study, we investigated the role of CWH43, a glycosylphosphatidylinositol-anchor biogenesis-related protein whose role in chemotherapy response has been unclear, in regulating redox balance and ferroptosis-associated vulnerability in colorectal cancer. Integrative analyses of clinical datasets, transcriptomic profiling, and functional experiments were performed using gain- and loss-of-function approaches in CRC cell models. We found that elevated CWH43 expression was associated with adverse clinical outcomes and reduced responsiveness to chemotherapy. Functionally, CWH43 attenuated ferroptosis-associated vulnerability by reducing intracellular reactive oxygen species and lipid peroxidation. Mechanistically, CWH43 promoted redox-adaptive programs involving activation of NRF2-dependent antioxidant signaling, upregulation of the cystine transporter SLC7A11, increased intracellular glutathione availability, and suppression of lipid peroxidation. Genetic or pharmacological disruption of SLC7A11 restored ferroptosis susceptibility and sensitized CWH43-overexpressing colorectal cancer cells to therapeutic stress, whereas ferrostatin-1 partially restored cell viability following erastin-containing treatments. Collectively, these findings identify CWH43 as a regulator of redox adaptation that modulates ferroptosis-associated vulnerability in colorectal cancer, providing insights into stress-adaptive survival mechanisms and chemotherapy resistance in cancer cells.

## 1. Introduction

Colorectal cancer (CRC) is among the most prevalent cancers globally. According to the latest GLOBOCAN 2024, it was estimated that there were more than 2.0 million new cases of CRC and over 918,000 deaths worldwide, making it the third most diagnosed cancer and the second leading cause of cancer death [1]. Clinically, systemic treatment of CRC is built around fluoropyrimidine-based combination chemotherapy. 5-fluorouracil (5-FU) combined with leucovorin forms the backbone of first-line regimens, most commonly delivered as FOLFOX (5-FU/leucovorin/oxaliplatin) or FOLFIRI (5-FU/leucovorin/irinotecan), with the triplet regimen FOLFOXIRI (5-FU, leucovorin, oxaliplatin, and irinotecan) reserved for selected fit patients requiring rapid tumor shrinkage [2,3]. These cytotoxic regimens are frequently combined with targeted agents chosen according to RAS/BRAF mutation status and tumor sidedness, including the antibody against vascular endothelial growth factor (VEGF), bevacizumab, and the antibodies against epidermal growth factor receptor (EGFR), cetuximab and panitumumab [2,3]. Despite significant progress in CRC treatment in recent years, chemotherapy resistance remains one of the main factors affecting patient prognosis. Chemotherapy resistance may result from several mechanisms, such as gene mutations, alterations in the tumor microenvironment, and increased intracellular drug efflux [4]. In the treatment of CRC, chemotherapy resistance remains a complex and challenging field, and future research should aim to identify resistance mechanisms and develop novel treatment strategies to enhance patient survival and quality of life [5].

CWH43 (Cell Wall Biogenesis 43 C-Terminal Homolog), also known as the PGAP2 (post-GPI attachment to proteins 2)-interacting protein, is a key protein involved in the biosynthesis of glycosylphosphatidylinositol (GPI) anchors, which are essential for anchoring specific membrane proteins to the cell membrane and are implicated in lipid remodeling [6,7,8,9]. GPI anchors covalently attach more than 150 distinct proteins, including enzymes, adhesion molecules, and cell-surface receptors, to the outer leaflet of the plasma membrane, and the lipid species generated by this remodeling process determine how these proteins partition into cholesterol- and sphingolipid-enriched membrane microdomains (lipid rafts) that organize receptor clustering and downstream signal transduction [10]. Because the integrity of these membrane microdomains influences the assembly of stress- and redox-signaling complexes at the cell surface, alterations in CWH43-dependent GPI lipid remodeling could plausibly extend beyond structural membrane anchoring to affect how CRC cells sense and adapt to oxidative and chemotherapeutic stress. Previous studies have suggested that CWH43 expression is dysregulated in colorectal cancer and may be associated with patient outcomes; however, its functional role in therapeutic response remains unclear [11,12]. Our previous research demonstrated that CWH43 is associated with attenuation of TTK (also known as monopolar spindle 1 kinase)-mediated cell cycle activity, suggesting a potential link to CRC progression [13]. However, whether CWH43 also contributes to chemotherapy resistance by modulating the redox-adaptive programs described above remains unknown. Further investigation into the involvement of CWH43 in chemotherapy resistance is crucial for developing more precise cancer therapies.

Ferroptosis, a novel form of programmed cell death driven by iron-dependent lipid peroxidation, has emerged as a promising target in cancer therapy [14]. Recent studies have shown that modulating specific signaling pathways can effectively induce ferroptosis across various cancer types. For example, ferroptosis induction via inhibition of glutathione peroxidase 4 (GPX4) has been demonstrated to sensitize cancer cells to chemotherapy [15]. Additionally, inhibition of solute carrier family 7 member 11 (SLC7A11), a key component of the cystine/glutamate antiporter system xc-, has been shown to trigger ferroptosis in CRC stem cells and suppress tumor progression [16]. By targeting the unique mechanisms underlying ferroptosis, researchers aim to address the challenges of drug resistance and enhance the efficacy of existing cancer therapies [17].

In this study, we investigated the role of CWH43 in chemotherapy resistance in CRC, hypothesizing that CWH43-dependent GPI lipid remodeling promotes a redox-adaptive phenotype that suppresses ferroptosis-associated vulnerability. Our findings reveal that elevated CWH43 expression correlates with reduced chemotherapy sensitivity in CRC patients, indicating a potential mechanism of chemoresistance associated with CWH43 levels. Furthermore, high CWH43 expression is associated with poorer survival outcomes in patients with advanced CRC, potentially involving modulation of reactive oxygen species (ROS) levels and enhancement of antioxidant defenses involving the nuclear factor erythroid 2-related factor 2 (NRF2)/SLC7A11 pathway. These results highlight the significance of CWH43 in influencing both treatment response and prognosis in CRC. Consequently, targeting CWH43 may represent a promising strategy for developing ferroptosis-inducing therapies, providing a novel approach for CRC treatment.

## 2. Results

### 2.1. Association of CWH43 Expression with Chemotherapy Response and Prognosis in CRC

**Figure 1:**
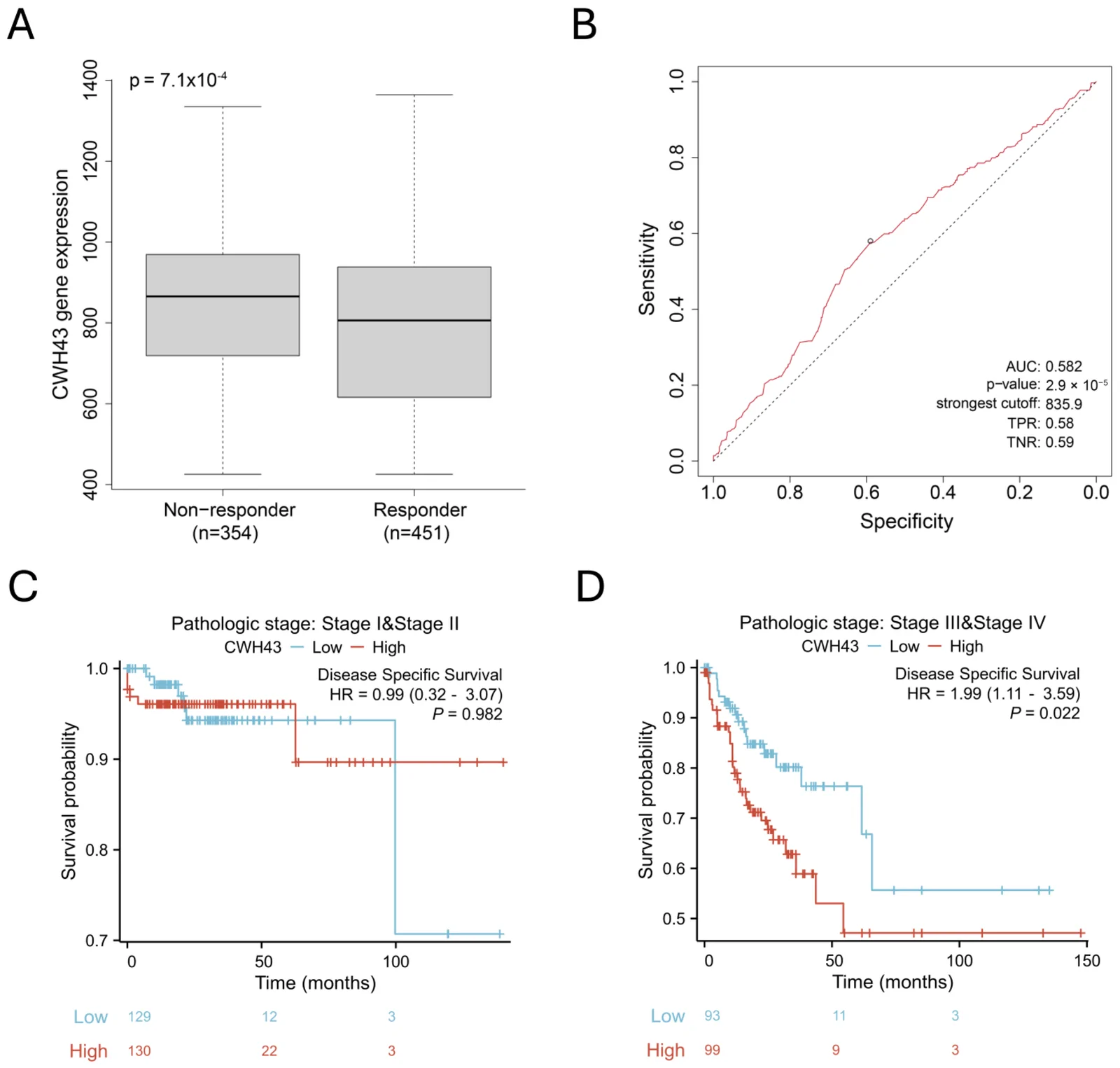
Association between CWH43 expression and chemotherapy response as well as disease-specific survival in colorectal cancer. (**A**) Comparison of CWH43 mRNA expression levels between chemotherapy responders and non-responders among colorectal cancer patients, showing significantly higher CWH43 expression in the non-responder group (*p* < 0.001, Mann–Whitney U test). (**B**) Receiver operating characteristic curve illustrating the discriminatory performance of CWH43 expression in predicting chemotherapy response, with an area under the curve (AUC) of 0.582 (*p* < 0.001). (**C**) Survival analysis in patients with stage I–II disease using TCGA COAD data. (**D**) Survival analysis in patients with stage III–IV disease, showing a significant association between elevated CWH43 expression and poorer disease-specific survival.

We previously proposed that CWH43 can regulate TTK to influence tumor progression in CRC [13], but the role of CWH43 in treatment response in CRC remains unclear. To clarify the impact of CWH43 on treatment efficacy, we used a receiver operating characteristic (ROC) plotter to determine the association of CWH43 expression with chemotherapy response in CRC. The ROC plotter database collected data on chemotherapy outcomes from 805 CRC patients, with 354 non-responders and 451 responders. The ROC plotter showed that CWH43 expression was significantly higher in non-responders compared to responders (*p* < 0.001) (Figure 1A). Furthermore, the analysis demonstrated that CWH43 expression showed a statistically significant but modest association with chemotherapy response in CRC, with an area under the curve (AUC) value of 0.582 (*p* < 0.001) (Figure 1B). In patients with advanced CRC, poor efficacy of chemotherapy is one of the significant reasons for an unfavorable prognosis [4]. To further investigate the clinical significance of CWH43 in advanced CRC, Kaplan–Meier survival analysis was conducted using colon adenocarcinoma (COAD) data from The Cancer Genome Atlas (TCGA). The results showed that in stage I–II patients, the expression of CWH43 was not associated with disease-specific survival (Figure 1C). Conversely, elevated CWH43 expression was strongly linked to poorer disease-specific survival in stage III–IV patients (Figure 1D). Collectively, CWH43 expression showed a statistically significant but modest association with chemotherapy response and was associated with poorer disease-specific survival in patients with stage III–IV CRC.

### 2.2. Validation of CWH43-Manipulated Cell Models and the Effect of CWH43 Overexpression on Chemotherapy Sensitivity

**Figure 2:**
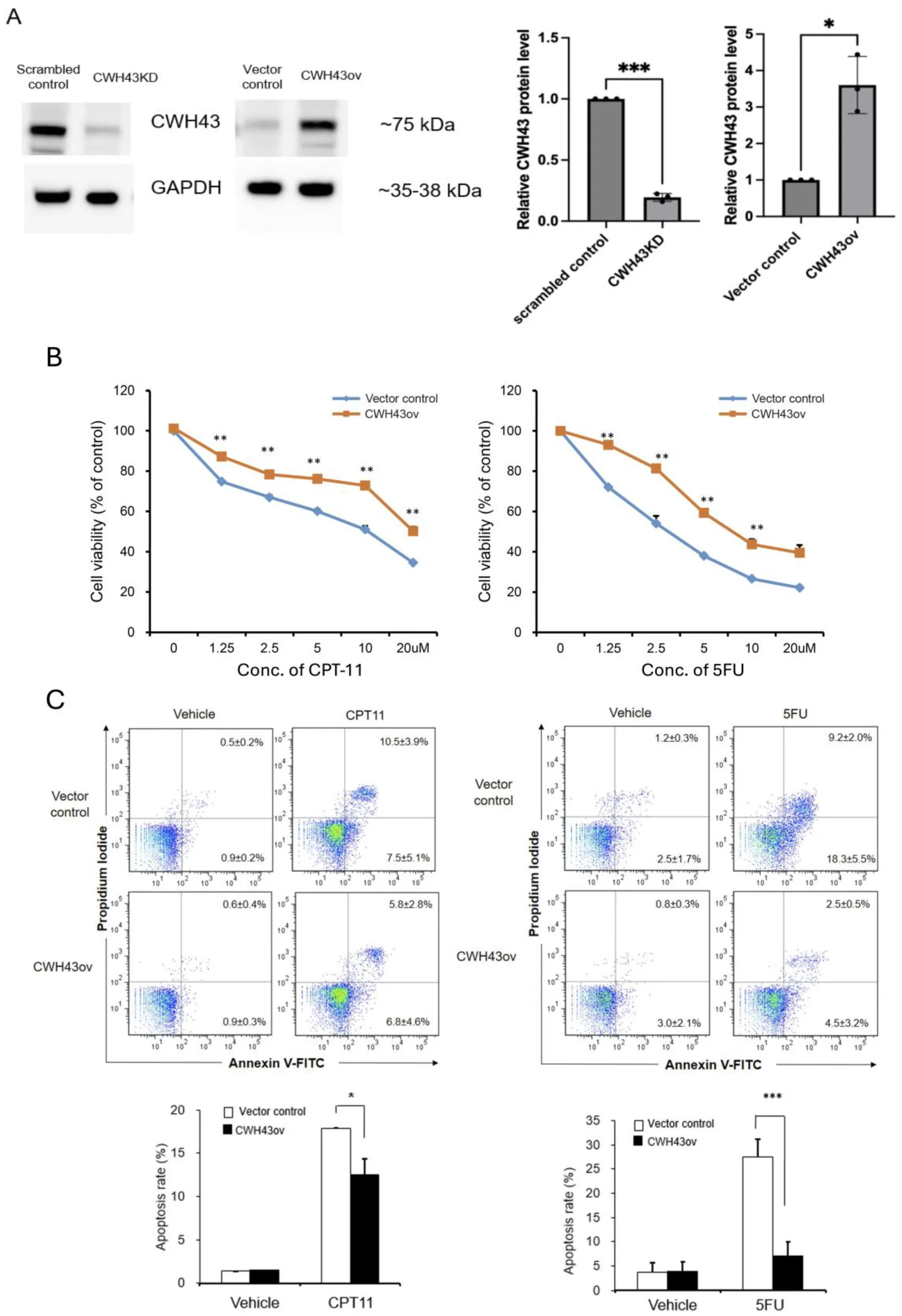
Validation of CWH43-manipulated colorectal cancer cell models and the effect of CWH43 overexpression on chemotherapy sensitivity. (**A**) Representative Western blots and densitometric quantification of CWH43 protein expression in scrambled-control and CWH43-knockdown cells and in vector control and CWH43-overexpressing cells. CWH43 expression was normalized to GAPDH and expressed relative to the corresponding control group. (**B**) Cell viability of vector control and CWH43-overexpressing HCT 116 cells following treatment with CPT-11 or 5-fluorouracil for 48 h, as assessed using the CCK-8 assay. (**C**) Flow cytometric analysis of apoptosis following CPT-11 or 5-fluorouracil treatment using Annexin V/propidium iodide staining. Data are presented as mean ± SD from three independent experiments. * *p* < 0.05, ** *p* < 0.01, and *** *p* < 0.001.

Western blot analysis confirmed that CWH43 protein expression was reduced in CWH43-knockdown cells and increased in CWH43-overexpressing cells relative to their respective controls (Figure 2A). We subsequently used these established cell models to investigate the effect of CWH43 expression on chemotherapy sensitivity [13]. To assess the in vitro cytotoxicity of chemotherapeutic agents on CWH43ov cells, we employed the Cell Counting Kit-8 (CCK-8) cell viability assay. CWH43ov cells were cultured for 48 h with five different concentrations of CPT-11 (1.25–20 μM) or 5-FU (1.25–20 μM). The results showed a significant increase in cell survival rate compared to the vector control (Figure 2B). Additionally, Annexin V/propidium iodide staining via flow cytometry showed that in the 20 μM CPT-11 treatment group, the apoptosis percentage in the vector control was 18%, whereas in CWH43ov cells, it decreased to 12.6%. In the 20 μM 5-FU treatment group, the apoptosis percentage in vector control cells was 27.5%, while in CWH43ov cells, it decreased to 7% (Figure 2C). These findings suggest that CWH43 overexpression diminishes CRC cell sensitivity to CPT-11 and 5-FU.

### 2.3. Functional Analysis of CWH43 in CRC Based on TCGA Data

**Figure 3:**
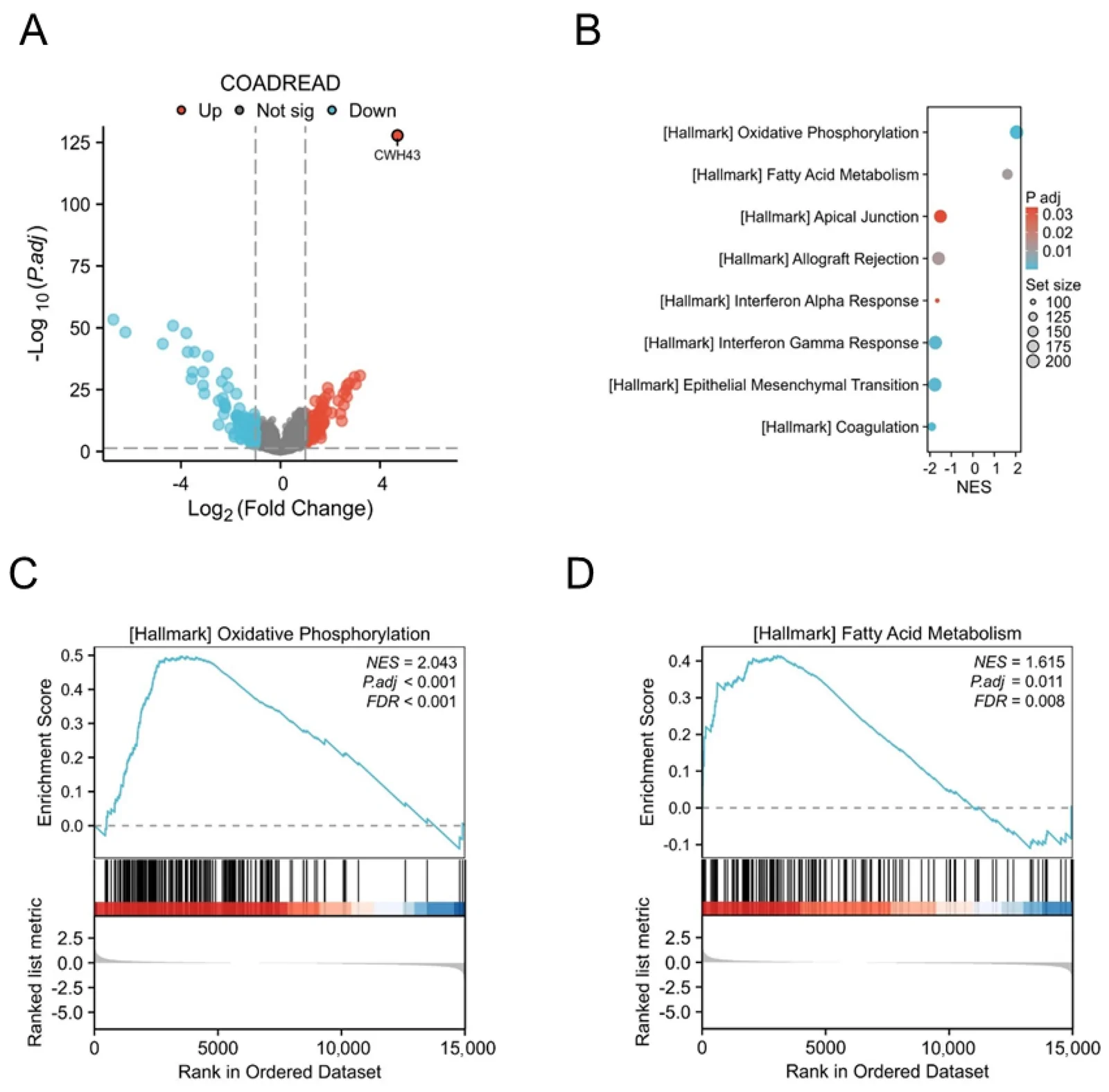
Functional enrichment analysis of transcriptomic differences associated with CWH43 expression in colorectal cancer. (**A**) Volcano plot showing differentially expressed genes between tumors with high and low CWH43 expression based on TCGA COADREAD data. (**B**) Bubble plot summarizing Gene Set Enrichment Analysis (GSEA) results for hallmark pathways. (**C**,**D**) Representative GSEA enrichment plots demonstrating positive associations between CWH43 expression and oxidative phosphorylation (**C**) and fatty acid metabolism (**D**). Genes ranked toward the left of the ordered dataset (red region of the bar) correspond to the high CWH43 expression group, whereas genes ranked toward the right (blue region) correspond to the low CWH43 expression group.

To investigate the role of CWH43 in enhancing chemotherapy resistance in CRC cells, we aimed to study its downstream regulatory mechanisms further. Using TCGA data from COAD and rectal adenocarcinoma (READ) cohorts, we divided patients into two groups based on median expression: high and low CWH43 expression. Subsequently, differential expression analysis between these two groups yielded 201 differentially expressed genes (DEGs), including 96 upregulated and 105 downregulated genes (Figure 3A). We performed a Gene Set Enrichment Analysis (GSEA) analysis based on the fold changes in DEGs and identified eight significantly enriched pathways (Figure 3B). The GSEA results showed that CWH43 was positively correlated with OXIDATIVE_PHOSPHORYLATION (Figure 3C) and FATTY_ACID_METABOLISM (Figure 3D), while negatively correlated with APICAL_JUNCTION, ALLOGRAFT_REJECTION, INTERFERON_ALPHA_RESPONSE, INTERFERON_GAMMA_RESPONSE, EPITHELIAL_MESENCHYMAL_TRANSITION, and COAGULATION (Figure S1).

### 2.4. CWH43 Influences the Status of Oxidative Phosphorylation and Intracellular ROS Levels

**Figure 4:**
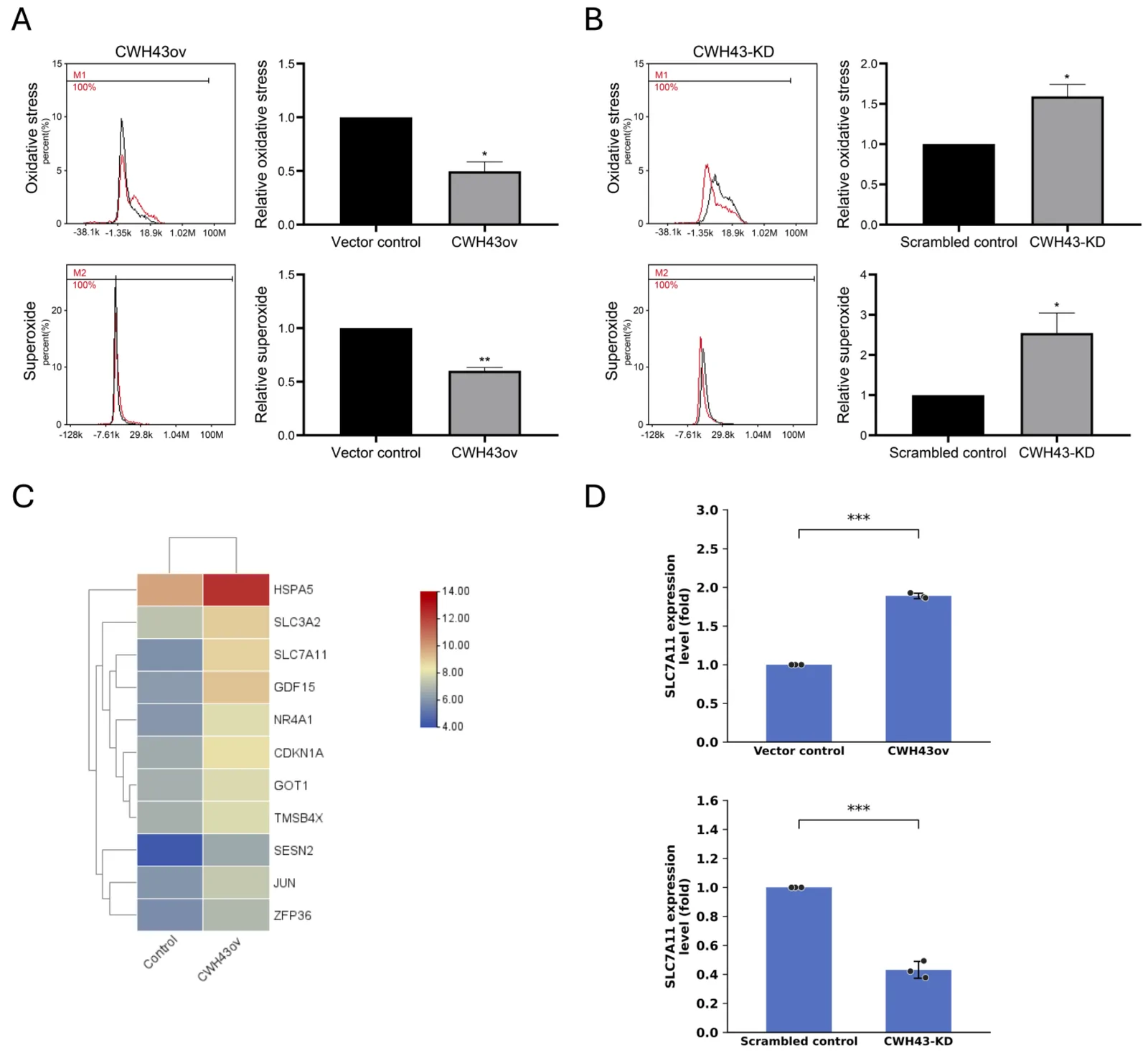
CWH43 expression is associated with altered intracellular redox status and expression of ferroptosis-related regulators in colorectal cancer cells. (**A**) Intracellular ROS and superoxide levels in vector control and CWH43-overexpressing (CWH43ov) HCT 116 cells. (**B**) ROS and superoxide levels in scrambled control and CWH43-knockdown (CWH43-KD) cells. (**C**) Heatmap showing the relative expression of ferroptosis-suppressive genes in CWH43ov cells compared to control cells, based on RNA sequencing analysis. (**D**) Quantitative reverse transcription polymerase chain reaction analysis of SLC7A11 expression in control, CWH43ov, and CWH43-KD cells. Data are presented as mean ± SD from at least three independent experiments. * *p* < 0.05, ** *p* < 0.01, *** *p* < 0.001.

Our GSEA results found that CWH43 may be involved in oxidative phosphorylation in CRC (Figure 3C), and oxidative phosphorylation can produce ROS due to electron transport chain (ETC) leakage [18]. Consequently, we utilized the CWH43ov and CWH43-KD cells to explore whether CWH43 influences intracellular ROS/superoxide levels. Results from the Total ROS/Superoxide Detection Kit indicated a reduction in ROS/superoxide levels in CWH43ov cells compared to the vector control (Figure 4A). At the same time, an increase was observed in CWH43-KD cells compared to the scrambled control (Figure 4B, Figure S2). These findings suggest that elevated CWH43 expression is associated with enhanced cellular antioxidant capacity, leading to decreased intracellular ROS/superoxide levels.

### 2.5. CWH43 Modulates the Ferroptosis Suppressors’ Expression Pattern

The antioxidant defense system is a critical mechanism for preventing ferroptosis [19]; thus, we used RNA-seq analysis to compare the expression of ferroptosis suppressors between vector control and CWH43ov cells. The heatmap results showed that the expression of 11 ferroptosis-suppressive genes was elevated in CWH43-overexpressing cells (Figure 4C). Among these candidates, SLC7A11 was selected for further investigation because it occupies a central upstream position in ferroptosis regulation by controlling cystine uptake, glutathione biosynthesis, and downstream antioxidant defenses. In addition, SLC7A11 is a direct target of the ferroptosis inducer erastin and has been reported to play critical roles in colorectal cancer stemness, therapeutic resistance, and ferroptosis susceptibility. Given its mechanistic relevance and therapeutic tractability, SLC7A11 was prioritized for subsequent validation and functional analyses. SLC7A11 was further examined using an RT-qPCR assay to assess its expression. The results showed that SLC7A11 expression was significantly upregulated in CWH43ov cells compared to control cells, while SLC7A11 mRNA levels decreased in CWH43-KD cells (Figure 4D).

### 2.6. CWH43 Expression Is Associated with NRF2-Dependent SLC7A11 Expression

**Figure 5:**
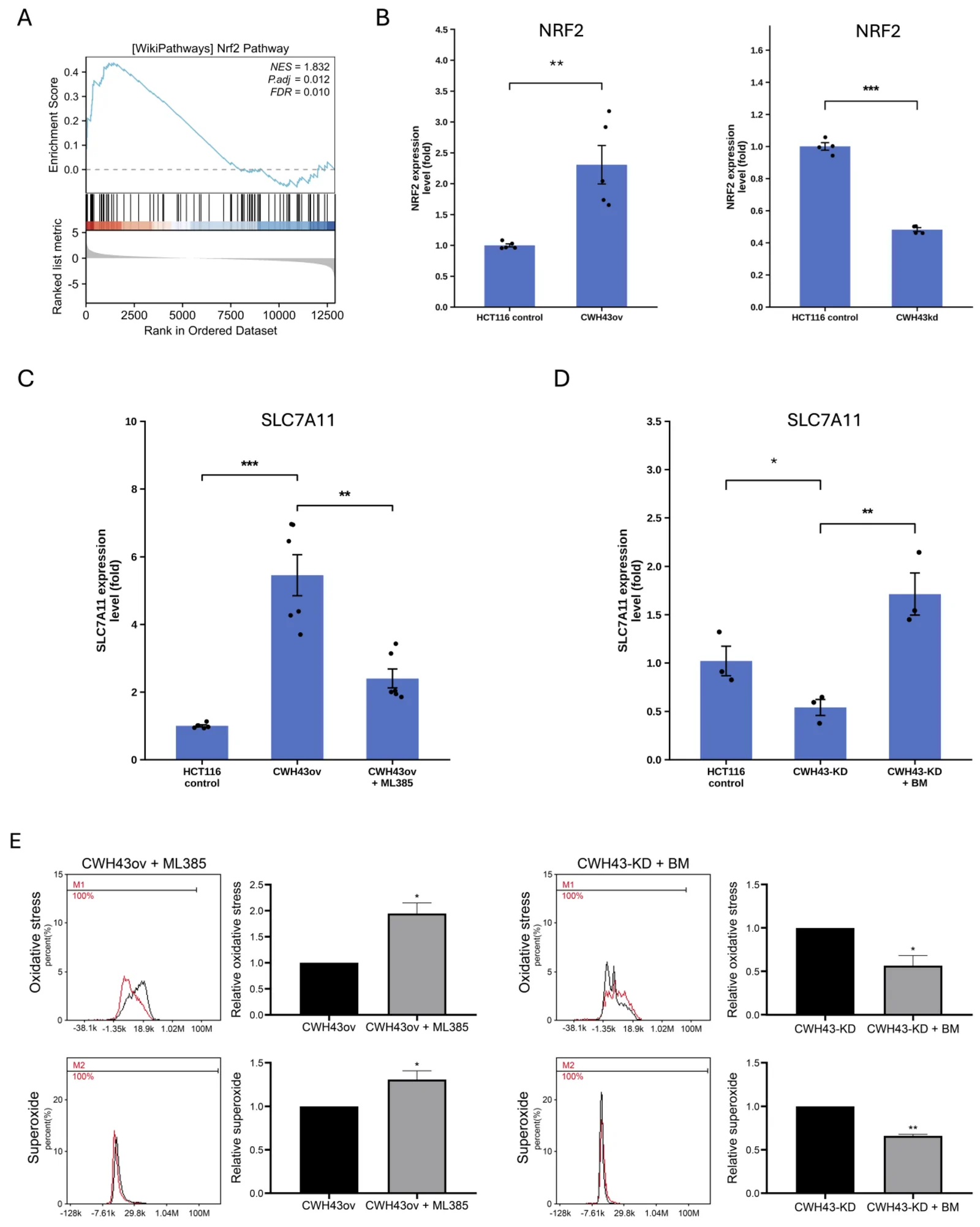
CWH43 expression is associated with NRF2 signaling activity and intracellular redox regulation. (**A**) GSEA showing enrichment of NRF2-related transcriptional signatures in CWH43-overexpressing cells. In the enrichment plot, the left side of the bar (red region) represents the CWH43 overexpression group, whereas the right side (blue region) represents the vector control group. (**B**) Quantitative RT-PCR analysis of NRF2 expression in control, CWH43ov, and CWH43-KD cells. (**C**) SLC7A11 expression in CWH43ov cells following treatment with the NRF2 inhibitor ML385. (**D**) SLC7A11 expression in CWH43-KD cells following treatment with the NRF2 activator bardoxolone methyl. (**E**) Intracellular ROS and superoxide levels following pharmacologic modulation of NRF2 activity. Data are presented as mean ± SD from at least three independent experiments. * *p* < 0.05, ** *p* < 0.01, *** *p* < 0.001.

To explore the potential mechanism by which CWH43 regulates SLC7A11, we performed GSEA on the RNA-seq results of CWH43 overexpression. The results showed a positive correlation between CWH43 and the NRF2 pathway (Figure 5A). Additionally, RT-qPCR assay results indicated that NRF2 expression was higher in CWH43ov cells than in the control group, while NRF2 expression was lower in CWH43-KD cells than in the control (Figure 5B). Since NRF2 is a potential upstream transcription factor of SLC7A11, we treated CWH43ov cells with ML385 (NRF2 inhibitor) and found a significant decrease in SLC7A11 expression (Figure 5C). On the other hand, after treating CWH43-KD cells with Bardoxolone methyl (an NRF2 activator), we observed a significant increase in SLC7A11 expression (Figure 5D). The above results indicate that CWH43 expression is associated with increased SLC7A11 expression in an NRF2-dependent manner.

The NRF2/SLC7A11 pathway maintains cellular redox balance and protects against oxidative stress. [20]. Thus, we aimed to explore whether CWH43 influences intracellular ROS/superoxide levels via the NRF2/SLC7A11 axis. The ROS/superoxide analysis showed that ROS/superoxide levels increased in CWH43ov cells treated with ML385 (NRF2 inhibitor) compared to the control group. In contrast, ROS/superoxide levels decreased in CWH43-KD cells treated with Bardoxolone methyl (NRF2 activator) (Figure 5E, Figure S3). These results suggest that CWH43 expression is associated with altered intracellular ROS/superoxide levels involving NRF2/SLC7A11-associated antioxidant signaling.

### 2.7. Ferroptosis-Associated Mechanisms Contribute to CWH43-Mediated Chemoresistance

**Figure 6:**
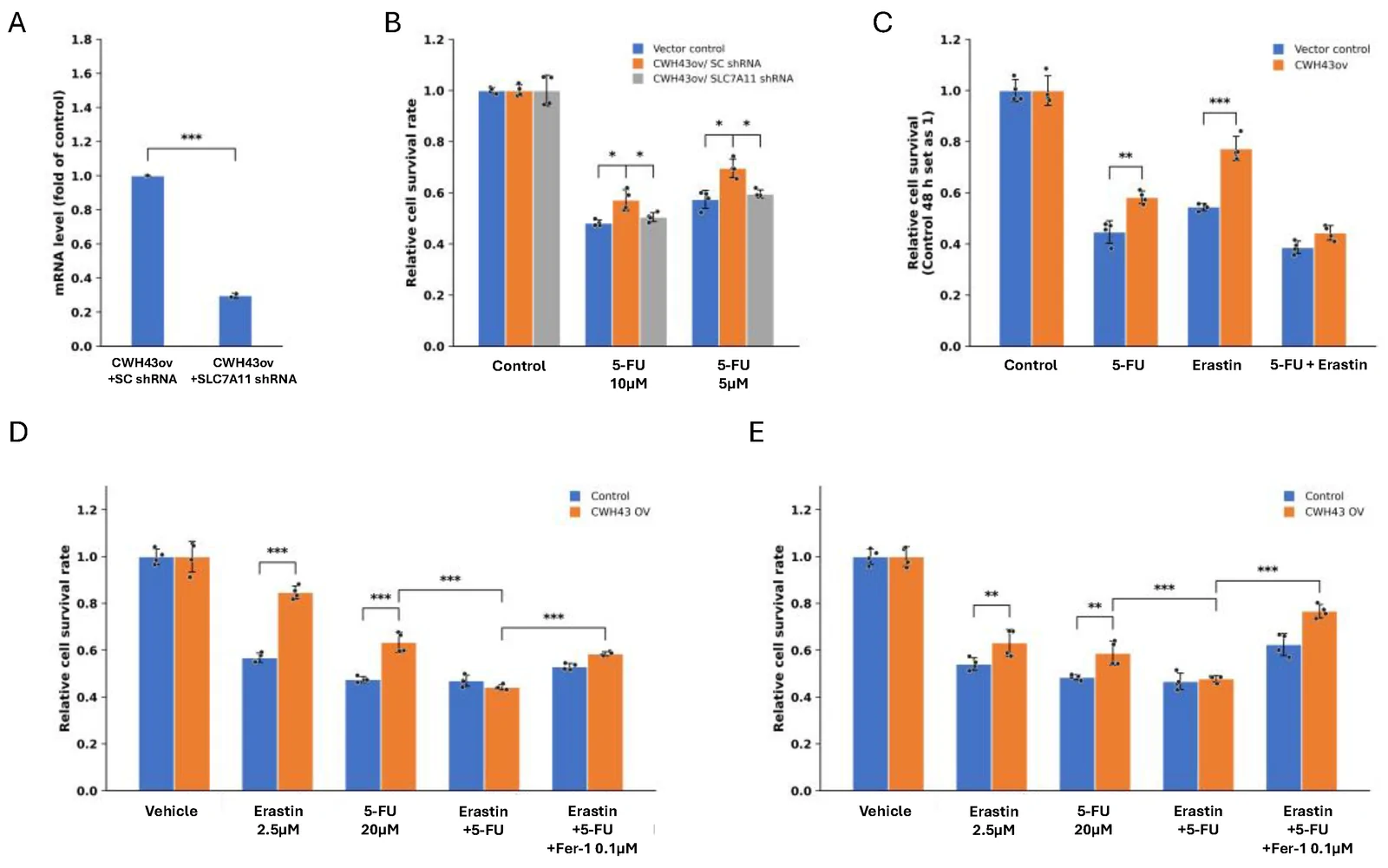
Ferroptosis-associated mechanisms contribute to CWH43-mediated chemoresistance in colorectal cancer cells. (**A**) Quantitative RT-PCR analysis confirming the knockdown efficiency of SLC7A11 in CWH43ov HCT 116 cells following transfection with SLC7A11-specific shRNA. SLC7A11 expression was normalized to the scrambled control (SC shRNA) group. (**B**) Cell viability of vector control, CWH43-overexpressing (CWH43ov), and CWH43ov/SLC7A11-knockdown cells following treatment with 5-fluorouracil (5-FU) at the indicated concentrations for 48 h. Cell viability was determined using the SRB assay. Silencing SLC7A11 partially reversed the 5-FU-resistant phenotype associated with CWH43 overexpression. (**C**) Cell viability of vector control and CWH43-overexpressing cells following treatment with 5-FU, erastin, or a combination of 5-FU and erastin for 48 h. CWH43 overexpression increased cell survival following either 5-FU or erastin treatment alone, whereas the survival advantage was markedly reduced by combined treatment with 5-FU and erastin. (**D**,**E**) DLD-1 (**D**) and HCT 116 (**E**) cells were treated with erastin, 5-FU, or the combination of erastin and 5-FU in the presence or absence of ferrostatin-1 (Fer-1). Cell viability was assessed using the SRB assay after 48 h of treatment. Erastin significantly reduced the survival advantage conferred by CWH43 overexpression under 5-FU treatment, whereas ferrostatin-1 partially restored cell viability, indicating that ferroptosis-associated mechanisms contribute to CWH43-mediated chemoresistance. Data are presented as mean ± SD from at least three independent experiments. * *p* < 0.05, ** *p* < 0.01, *** *p* < 0.001.

Ferroptosis induction has been demonstrated to reverse chemotherapy resistance and represents a novel therapeutic strategy for overcoming drug resistance in diverse cancer types [21]. To determine whether SLC7A11 contributes to the chemoresistant phenotype associated with CWH43 overexpression, SLC7A11 expression was silenced in CWH43ov cells using a specific short hairpin RNA (shRNA) (Figure 6A). Sulforhodamine B (SRB) cell viability assays demonstrated that SLC7A11 knockdown significantly reduced the survival of CWH43ov cells following 5-FU treatment, supporting the involvement of SLC7A11 in the chemoresistant phenotype associated with CWH43 overexpression (Figure 6B). To further investigate the involvement of ferroptosis-associated mechanisms, cells were treated with erastin, a ferroptosis inducer that inhibits SLC7A11 activity. Consistent with the chemoresistant phenotype, CWH43ov cells exhibited significantly higher viability than vector control cells following treatment with either 5-FU or erastin alone (Figure 6C). However, combined treatment with 5-FU and erastin markedly reduced the survival advantage conferred by CWH43 overexpression, suggesting that ferroptosis induction sensitizes CWH43-overexpressing cells to chemotherapy (Figure 6C). To further validate the contribution of ferroptosis to CWH43-mediated chemoresistance, DLD-1 and HCT 116 cells were treated with 5-FU and erastin in the presence or absence of the ferroptosis inhibitor ferrostatin-1. As expected, CWH43-overexpressing cells displayed increased resistance to both 5-FU and erastin treatment compared with control cells (Figure 6D,E). Notably, erastin significantly reduced the survival advantage of CWH43-overexpressing cells under 5-FU treatment, whereas ferrostatin-1 partially restored cell viability in both cell lines. These findings suggest that suppression of ferroptosis-associated vulnerability contributes, at least in part, to the chemoresistant phenotype induced by CWH43 overexpression (Figure 6D,E).

### 2.8. CWH43 Activates Antioxidant Defense Programs and Reduces Ferroptosis-Associated Lipid Peroxidation

**Figure 7:**
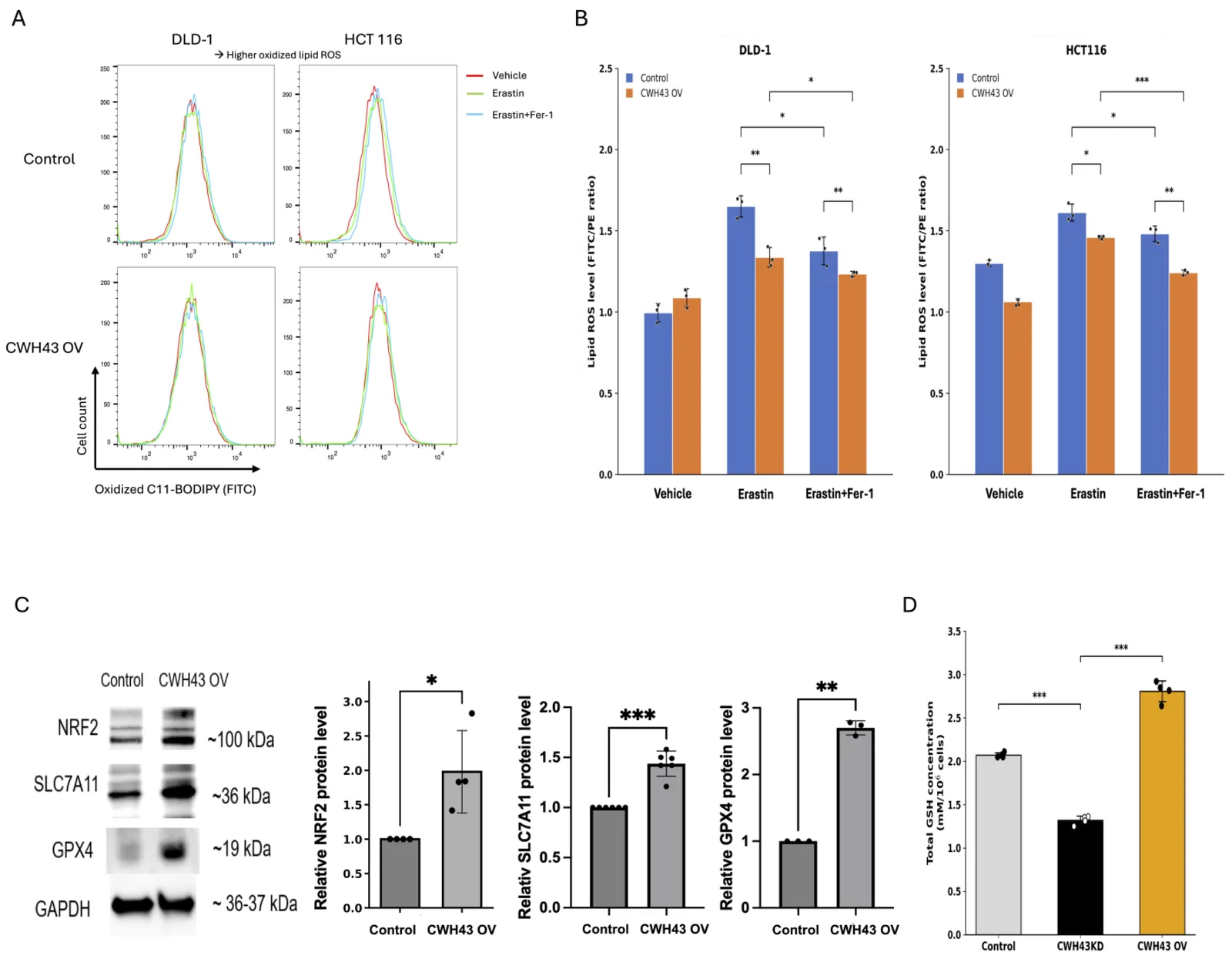
CWH43 expression is associated with enhanced antioxidant defense and reduced lipid peroxidation in colorectal cancer cells. (**A**) Representative flow-cytometric histograms of C11-BODIPY 581/591 fluorescence in vector control and CWH43ov DLD-1 and HCT 116 cells treated with vehicle, erastin (5 μM), or erastin plus ferrostatin-1 (Fer-1; 0.1 μM) for 6 h. A rightward shift in oxidized C11-BODIPY fluorescence indicates increased lipid peroxidation. (**B**) Quantification of lipid reactive oxygen species (ROS), expressed as the ratio of oxidized FITC-channel fluorescence to reduced PE-channel fluorescence (FITC/PE ratio). (**C**) Representative Western blot images and densitometric analyses of NRF2, SLC7A11, and GPX4 protein expression in vector control and CWH43ov DLD-1 cells. Protein expression was normalized to the corresponding GAPDH loading control and expressed relative to the vector-control group. The displayed blots are representative of the independent biological experiments used for quantification. NRF2 was quantified from four independent experiments, GPX4 was quantified from three independent experiments, and SLC7A11 was quantified from six independent experiments. (**D**) Intracellular total glutathione (T-GSH) concentrations in vector control, CWH43-KD, and CWH43ov DLD-1 cells, normalized to cell number. Data in (**B**–**D**) are presented as mean ± SD from independent biological experiments; * *p* < 0.05, ** *p* < 0.01, and *** *p* < 0.001.

To further investigate the mechanism underlying ferroptosis-associated vulnerability regulated by CWH43, lipid peroxidation was evaluated using C11-BODIPY 581/591 staining. Flow cytometric analysis demonstrated that erastin treatment increased lipid ROS accumulation in both DLD-1 and HCT 116 cells. Notably, CWH43 overexpression consistently attenuated erastin-induced lipid peroxidation. Ferrostatin-1 co-treatment partially suppressed the erastin-induced increase in lipid peroxidation (Figure 7A,B). Furthermore, CWH43 overexpression increased the protein expression levels of NRF2, SLC7A11, and GPX4, key regulators of cellular antioxidant defense and ferroptosis resistance (Figure 7C). Consistently, total glutathione levels were significantly reduced in CWH43-knockdown cells and significantly elevated in CWH43-overexpressing cells (Figure 7D). These findings suggest that CWH43 expression is associated with activation of an NRF2/SLC7A11-related antioxidant program, increased glutathione availability, reduced lipid peroxidation, and diminished ferroptosis-associated vulnerability in CRC cells.

### 2.9. CWH43 Expression Is Associated with Reduced Mitochondrial Depolarization Under Erastin-Induced Stress

To determine whether CWH43 expression is associated with changes in mitochondrial membrane potential, we performed JC-1 staining followed by flow cytometric analysis. DLD-1 and HCT 116 cells with stable CWH43 knockdown or overexpression, together with their respective controls, were treated with 5 μM erastin in the presence or absence of 0.1 μM ferrostatin-1 (Fer-1). The JC-1 green/red fluorescence ratio was used as an indicator of mitochondrial depolarization, with a higher ratio representing a greater loss of mitochondrial membrane potential.

**Figure 8:**
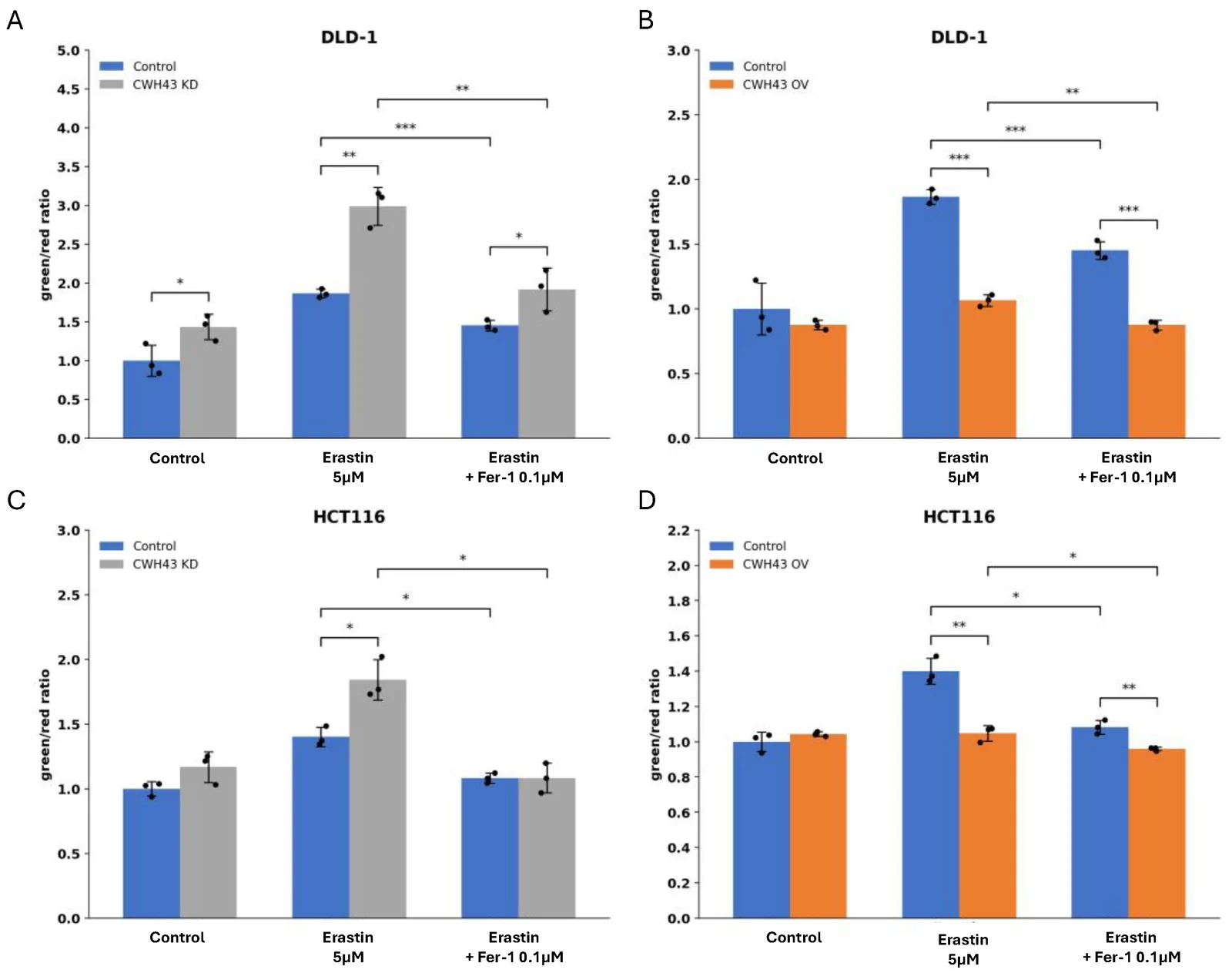
CWH43 expression is associated with reduced mitochondrial depolarization under erastin-induced stress. DLD-1 (**A**,**B**) and HCT 116 (**C**,**D**) cells were treated with erastin (5 μM) alone or in combination with ferrostatin-1 (Fer-1, 0.1 μM). Mitochondrial membrane potential was assessed by JC-1 staining and flow cytometry and is expressed as the ratio of green (monomeric, low-potential) to red (J-aggregate, high-potential) fluorescence intensity, with a higher ratio indicating mitochondrial depolarization. Data are presented as mean ± SD from three independent experiments. * *p* < 0.05, ** *p* < 0.01, *** *p* < 0.001.

In DLD-1 cells, CWH43 knockdown significantly increased the basal JC-1 green/red ratio relative to the scrambled control and further increased the ratio following erastin treatment. Fer-1 co-treatment partially attenuated the erastin-associated increase in mitochondrial depolarization in CWH43-knockdown cells (Figure 8A). Conversely, CWH43 overexpression attenuated the increase in the JC-1 green/red ratio induced by erastin. Fer-1 co-treatment further reduced mitochondrial depolarization and diminished the difference between vector-control and CWH43-overexpressing cells (Figure 8B). Similar treatment-dependent patterns were observed in HCT 116 cells, although the basal effect of CWH43 knockdown was less pronounced than that observed in DLD-1 cells (Figure 8C,D).

Collectively, these findings indicate that CWH43 expression is associated with reduced mitochondrial depolarization, particularly under erastin-induced stress. Together with the observed changes in intracellular ROS and lipid peroxidation, the JC-1 results support an association between CWH43-mediated redox adaptation and the maintenance of mitochondrial membrane potential during ferroptosis-related stress. However, JC-1 reflects mitochondrial membrane potential and does not independently establish ferroptotic cell death.

## 3. Discussion

CRC remains a major cause of cancer-related mortality, with chemotherapy resistance posing a substantial barrier to effective treatment [22,23]. In this study, we investigated the association between CWH43 expression, chemotherapy response, and stress-adaptive cell survival programs in CRC. Analysis using ROCplot.org showed that elevated CWH43 expression was associated with reduced chemotherapy responsiveness (AUC = 0.582). However, the modest AUC indicates limited discriminatory performance when CWH43 is used alone. Therefore, CWH43 should be considered a candidate response-associated biomarker that requires further validation rather than an established predictor of chemotherapy response [24,25]. Consistently, survival analysis based on TCGA clinical data has demonstrated that high CWH43 expression correlates with poorer disease-specific survival in advanced-stage CRC (stages III–IV), but not in early-stage disease, indicating a context-dependent role for CWH43 in disease progression and therapeutic outcome [26]. This stage-restricted association may be biologically plausible. Patients with advanced-stage disease are more likely to receive systemic chemotherapy and to experience greater treatment-related selective pressure. Advanced tumors may also encounter more pronounced hypoxic, nutritional, and oxidative stress within the tumor microenvironment [27]. These conditions could increase the biological relevance of CWH43-associated redox adaptation and chemoresistance. Nevertheless, this interpretation remains hypothesis-generating and requires validation in independent, treatment-annotated clinical cohorts.

Functional studies in CRC cell models further showed that CWH43 overexpression was associated with reduced sensitivity to commonly used chemotherapeutic agents, including CPT-11 and 5-fluorouracil. These findings suggest that CWH43 may facilitate chemoresistance through cellular stress-adaptive mechanisms rather than by directly altering drug transport or metabolism [23,28,29,30]. In line with this notion, transcriptomic analyses revealed that CWH43 expression was positively associated with oxidative phosphorylation and fatty acid metabolism pathways, both of which have been implicated in chemoresistance and redox homeostasis [31,32,33,34,35]. Notably, the degree of CWH43-associated protection differed between the two agents, being more pronounced against CPT-11 than against 5-FU. CPT-11 (irinotecan), a topoisomerase I inhibitor, induces replication-associated DNA damage and has also been reported to alter mitochondrial metabolism and increase reactive oxygen species in CRC cells [36]. In contrast, 5-FU acts primarily through thymidylate synthase inhibition and the incorporation of its metabolites into RNA and DNA [37]. These distinct primary mechanisms raise the possibility that oxidative and mitochondrial stress may contribute differently to the cellular responses to these agents. Accordingly, CWH43-associated redox adaptation may influence CPT-11 and 5-FU sensitivity to different extents. However, the present experiments were not designed to directly compare the drug-specific contributions of oxidative stress, and this interpretation therefore remains hypothesis-generating.

Mitochondrial oxidative phosphorylation is a major source of intracellular ROS due to electron leakage from the electron transport chain [18,38]. Excessive ROS accumulation can induce multiple forms of regulated cell death, including ferroptosis, which is characterized by iron-dependent lipid peroxidation [14,19,39]. Our data demonstrated that CWH43 overexpression was associated with reduced intracellular ROS and superoxide levels, whereas CWH43 knockdown resulted in ROS elevation, suggesting that CWH43 expression is linked to enhanced antioxidant capacity. These findings support a role for CWH43 in promoting redox-adaptive states that may enable CRC cells to better tolerate chemotherapy-induced oxidative stress. Our transcriptomic data showed that CWH43-high tumors displayed coordinate enrichment of oxidative phosphorylation and fatty acid metabolism [40]. Mechanistically, oxidative phosphorylation supplies the mitochondrial ROS and fatty acid metabolism supplies the peroxidizable phospholipid substrates that together drive iron-dependent lipid peroxidation [41]. The enrichment of oxidative phosphorylation and fatty acid metabolism, together with the observed changes in intracellular ROS, provided a biological rationale for investigating ferroptosis-related mechanisms. Mitochondrial redox activity and lipid metabolism can influence oxidative stress and the availability of peroxidizable phospholipids, respectively [42]. We therefore focused our subsequent experiments on ferroptosis. Nevertheless, these findings do not exclude the possible involvement of other forms of regulated cell death, which were not comprehensively evaluated in this study. In addition, the positive association between CWH43 expression and oxidative phosphorylation is also of interest in light of evidence that cancer stem cells in several tumor types preferentially rely on OXPHOS rather than aerobic glycolysis to sustain their stemness, therapy resistance, and tumor-initiating capacity [43]. Given that CWH43-high CRC cells display reduced chemotherapy sensitivity and enrichment of an OXPHOS-related transcriptional signature, CWH43 may favor a metabolic state resembling that of OXPHOS-dependent, therapy-resistant cancer stem-like cells, rather than simply protecting bulk tumor cells from oxidative damage. This raises the possibility that CWH43-driven GPI lipid remodeling could influence stem-like plasticity in CRC. It should be emphasized that this interpretation is based on a transcriptomic association (enrichment of the OXPHOS pathway) rather than on direct functional evidence obtained in a cancer stem cell model, and should therefore be regarded as a speculative observation pending future investigation using stemness markers and functional assays.

Because NRF2 is a well-established master regulator of the cellular antioxidant response to ROS [44], we next asked whether the reduced-ROS, antioxidant phenotype of CWH43-overexpressing cells involved activation of NRF2 signaling. Gene set enrichment analyses indicated activation of NRF2-dependent transcriptional programs in CWH43-overexpressing cells. NRF2 is a central regulator of cellular antioxidant responses and has been shown to limit ferroptosis and promote cancer cell survival under stress conditions [45,46,47]. Pharmacologic modulation of NRF2 activity altered SLC7A11 expression and intracellular ROS levels, supporting a functional connection between CWH43 expression, NRF2 signaling, and redox regulation. While these data support the involvement of an NRF2/SLC7A11–associated antioxidant program, additional redox-regulated or metabolic pathways may also contribute to CWH43-associated chemoresistance. Given this established link between NRF2 activation and suppression of ferroptosis, we then examined SLC7A11, a direct transcriptional target of NRF2 and a central ferroptosis-suppressive regulator identified among the ferroptosis-suppressive genes revealed by RNA-seq. SLC7A11 was selected as the primary candidate because it occupies a central position linking cystine metabolism, glutathione biosynthesis, and ferroptosis regulation [48]. Consistent with this redox-adaptive phenotype, CWH43 overexpression was associated with increased expression of ferroptosis-suppressive regulators, including SLC7A11, a critical component of the cystine/glutamate antiporter system xc− that supports glutathione synthesis and protects against lipid peroxidation [16,49,50,51]. Although NRF2, SLC7A11, and GPX4 protein expression were evaluated in the CWH43-overexpression model, the observed alterations in glutathione levels in both CWH43-overexpressing and CWH43-knockdown cells further support the role of CWH43 in regulating antioxidant capacity and ferroptosis-associated vulnerability.

Ferroptosis has emerged as an attractive therapeutic vulnerability in drug-resistant cancers [21,52,53]. In this study, genetic or pharmacologic disruption of SLC7A11 function increased chemotherapy sensitivity in CWH43-overexpressing cells, suggesting that CWH43-high CRC cells may exhibit reduced susceptibility to ferroptosis-associated stress. These findings are consistent with prior studies demonstrating that suppression of NRF2 signaling or ferroptosis-protective mechanisms can sensitize cancer cells to anticancer therapies [54,55]. Rather than acting as a direct inhibitor of ferroptosis, CWH43 may contribute to a cellular state in which ferroptosis-related vulnerability is diminished, thereby facilitating survival under chemotherapeutic pressure.

Another notable finding was the negative correlation between CWH43 expression and the EMT hallmark signature, which was seemingly counterintuitive. Since EMT is classically linked to invasiveness, stemness, and chemoresistance, phenotypes are expected to accompany rather than oppose the poor prognosis and reduced chemosensitivity seen in CWH43-high tumors. However, chemoresistance and EMT are not obligatorily coupled. Redox- and metabolism-driven resistance, such as the OXPHOS/antioxidant program described here, can act largely independently of EMT signaling [56], and epithelial or hybrid epithelial/mesenchymal states are increasingly recognized as alternative routes to drug tolerance that do not require full mesenchymal conversion [57]. The EMT signature measured here also reflects bulk transcriptomic state in treatment-naïve tumors and does not exclude chemotherapy-induced EMT activation later in treatment or at relapse. Together, these points suggest CWH43 may drive a chemoresistant, redox-adapted phenotype through a largely EMT-independent mechanism rather than classical mesenchymal transformation. Direct assessment of EMT markers and invasive/migratory behavior in CWH43 perturbation cells will be needed to test this interpretation and reconcile these seemingly opposing programs in CWH43-high CRC.

Interestingly, our findings reveal a potentially context-dependent role of CWH43 in colorectal cancer biology. In our previous study, CWH43 was identified as a tumor suppressor that inhibits CRC progression through negative regulation of TTK-mediated cell-cycle signaling. In contrast, the present study demonstrates that elevated CWH43 expression is associated with enhanced antioxidant capacity, reduced ferroptosis-associated vulnerability, and decreased sensitivity to chemotherapeutic stress. These observations suggest that the biological functions of CWH43 may vary according to cellular context and environmental conditions. The apparent discrepancy may reflect distinct biological roles of CWH43 in basal proliferative regulation versus stress-adaptive responses during chemotherapy exposure. While CWH43 may suppress tumor growth under basal conditions, it may simultaneously facilitate adaptive survival programs under chemotherapy-induced oxidative stress through activation of NRF2/SLC7A11-associated antioxidant signaling. These findings highlight the complexity of CWH43 biology and suggest that its functional impact may depend on the balance between proliferative control and stress adaptation. Further studies are required to clarify how these distinct functions are coordinated during colorectal cancer progression and treatment.

Several limitations of this study should be acknowledged. First, the mechanistic interpretation is primarily based on in vitro cell models and retrospective bioinformatic analyses. In vivo studies and prospectively collected, treatment-annotated clinical cohorts will therefore be required to establish the therapeutic relevance of CWH43-associated redox adaptation and to evaluate its potential as a predictive biomarker of chemotherapy response. Second, we did not directly assess CWH43 function in dedicated cancer stem-cell models or confirm ferroptotic cell death using additional orthogonal approaches. Studies incorporating stemness markers, spheroid formation, tumor-initiating capacity, and complementary ferroptosis assays will be needed to further define the role of CWH43 in chemotherapy resistance. Third, SLC7A11 knockdown was evaluated only in CWH43-overexpressing cells. Although SLC7A11 depletion attenuated the survival advantage associated with CWH43 overexpression, the current experimental design does not establish whether this effect is selective for the CWH43-overexpressing background. Future studies should therefore evaluate SLC7A11 depletion in vector-control cells to determine context specificity. Finally, the pharmacological interaction between 5-FU and erastin was not formally evaluated using a complete dose–response matrix. Ferrostatin-1 was used as a mechanistic rescue agent rather than as a component of a formal drug-combination analysis. Future dose-matrix experiments and quantitative synergy analyses will be required to distinguish additive from synergistic interactions between 5-FU and erastin.

## 4. Materials and Methods

### 4.1. Bioinformatic Analyses

The ROC plotter (https://rocplot.org/) was utilized to evaluate the association between CWH43 gene expression and therapy response in a cohort of 805 CRC patients (354 non-responders and 451 responders) classified according to Response Evaluation Criteria in Solid Tumors (RECIST). Receiver operating characteristic (ROC) curves and the corresponding area under the curve (AUC) values were generated to assess biomarker performance [24,25]. Patients were divided into two groups for survival analysis based on the median mRNA expression level, and the log-rank *p* value and the hazard ratio (HR) were calculated. RNA sequencing data for COAD (TPM), processed via the Spliced Transcripts Alignment to a Reference (STAR) workflow, and associated clinical information were obtained from the TCGA database (https://portal.gdc.cancer.gov). Patient survival data were extracted from a published study [26]. Cox regression analysis was conducted using the survival [3.3.1] package, and the results were visualized with survminer and ggplot2. A *p*-value < 0.05 was considered statistically significant.

### 4.2. Chemicals, Reagents, and Cell Culture

Fluorouracil (5-FU), irinotecan (CPT-11), and erastin were purchased from SelleckChem (Hsinchu, Taiwan). Stock solutions were prepared by dissolving the compounds in dimethyl sulfoxide (DMSO) (Sigma Aldrich, St. Louis, MO, USA) and stored at −20 °C. Before use, stock solutions were diluted in a sterile medium to the required concentrations. Human CRC cell lines HCT 116 (CCL-247) and DLD-1 (CCL-221) were obtained from the American Type Culture Collection (ATCC, Manassas, VA, USA). Both cell lines were maintained in RPMI 1640 medium supplemented with 10% fetal bovine serum, 1% penicillin/streptomycin, and 2 mM L-glutamine (Gibco, Thermo Fisher Scientific, Waltham, MA, USA). All cultures were maintained at 37 °C in a humidified incubator with 5% CO_2_. Sub-culturing or experiments were performed when the cells reached a confluence of less than 80% to ensure optimal cell health and experimental consistency. All experiments were performed using cells within passage numbers 5–15 after thawing. Cells were routinely tested for mycoplasma contamination every two weeks using a PCR-based mycoplasma detection kit, and only mycoplasma-negative cultures were used for the experiments.

### 4.3. Gene Expression Manipulation in CRC Cells

Knockdown CWH43 (CWH43-KD) and CWH43 overexpressing (CWH43ov) cells were generated as described in a previous study [13]. Briefly, CWH43-KD cells were generated by transfecting HCT 116 or DLD-1 cells with a CWH43-targeting shRNA (TRCN0000417738 and TRCN0000429495; National RNAi Core Facility, Academia Sinica, Taiwan) or a nontarget shRNA as the scrambled control, followed by two weeks of puromycin selection to establish stable clones. CWH43ov cells were generated by introducing the pCMV6-Entry-CWH43 plasmid (RC224386, OriGene Technologies, Rockville, MD, USA) into HCT 116 and DLD-1 cells via electroporation. Knockdown and overexpression efficiency were confirmed by RT-qPCR analysis. For SLC7A11 knockdown experiments, short hairpin RNA (shRNA; TRCN0000043124) targeting human SLC7A11 (NM_014331) was obtained from the National RNAi Core Facility at Academia Sinica, Taiwan. The target sequence for SLC7A11 was GCACCCTTTGACAATGATAAT. The scrambled control or SLC7A11 shRNA was transfected into CWH43ov cells using a Neon microporator (Life Technologies, Carlsbad, CA, USA) with a pipette-type system. Transfection was performed with a single pulse at 1.4 kV for 20 ms. Knockdown efficiency was confirmed by assessing SLC7A11 expression using RT-qPCR.

### 4.4. Cell Viability Assay

Cell viability was assessed using the Cell Counting Kit-8 (CCK-8) (Dojindo, Mashiki, Japan) and a sulforhodamine B (SRB)-based in vitro toxicology assay kit (TOX6, Sigma-Aldrich, St. Louis, MO, USA). For the CCK-8 assay, cells (7 × 10^3^ cells/well) were seeded into 96-well plates and cultured for 16 h. Cells were treated with varying doses of chemotherapy drugs for 48 h, followed by incubation with CCK-8 solution for two hours. The optical density (OD) at 450 nm was measured using a microplate reader (Bio-Rad Laboratories, Hercules, CA, USA). The SRB assay was used as a complementary endpoint in the ferroptosis-related experiments because it estimates adherent cellular biomass based on total cellular protein and is less directly dependent on cellular dehydrogenase-mediated reducing activity than the CCK-8 assay. Because CCK-8 and SRB measure different biological properties, results obtained using these assays were interpreted within their respective experiments and were not directly compared across assays.

For the SLC7A11-knockdown experiments, CWH43-overexpressing HCT 116 cells transfected with scrambled-control or SLC7A11-targeting shRNA were seeded in 96-well plates at a density of 7 × 10^3^ cells/well. For the pharmacological experiments, vector-control and CWH43-overexpressing DLD-1 and HCT 116 cells were seeded at the same density. After overnight attachment, cells were treated with 5-FU, erastin, ferrostatin-1, or the indicated combinations for 48 h.

Following treatment, adherent cells were fixed with 10% (wt/vol) trichloroacetic acid at 4 °C overnight and stained with 0.4% (wt/vol) SRB for 30 min at room temperature. Excess dye was removed by washing with 1% acetic acid, and the plates were air-dried. Protein-bound dye was solubilized in 10 mM Tris base, and absorbance was measured at 515 nm using a microplate reader. Absorbance values were normalized to the corresponding vehicle-control group and expressed as relative cellular biomass.

Each condition was assessed in triplicate wells within each experiment. All experiments were independently repeated at least three times, and the values obtained from the independent biological experiments were used for statistical analysis.

### 4.5. Annexin V/PI Double Stain

At 48 h post-treatment with chemotherapy drugs, cells were harvested by trypsinization and washed twice with cold phosphate-buffered saline (0.15 mol/L, pH 7.2). The cells were centrifuged at 300× *g* for five minutes, and the supernatant was discarded. The resulting pellet was resuspended in binding buffer to a final density of 1.0 × 10^5^ to 1.0 × 10^6^ cells/mL. For staining, 100 μL of the cell suspension was incubated with two μL of FITC-conjugated annexin V (Pharmingen) and two μL of PI (Pharmingen) for 15 min at room temperature in the dark. After staining, 400 μL of binding buffer was added to each tube. Samples were analyzed using a BD FACS Verse™ Flow Cytometer (BD Biosciences, San Jose, CA, USA), and data were processed with FlowJo software (version 1.54t; National Institutes of Health, Bethesda, MD, USA).

### 4.6. Functional Enrichment Analysis

Differential gene expression analysis was performed using the edgeR [3.38.2] package on TCGA COADREAD data to compare high and low CWH43 expression groups [58]. Differentially expressed genes (DEGs) were identified based on a threshold of |LogFC| ≥ 1 and p.adj ≤ 0.05. Gene Set Enrichment Analysis (GSEA) was conducted using the clusterProfiler [4.4.4] package to identify significantly enriched pathways and biological processes associated with the DEGs [59]. The results were visualized using the ggplot2 [3.3.6] package for enhanced graphical representation. Hallmark gene sets were considered significantly enriched when the nominal *p* value < 0.05 and the false discovery rate q-value < 0.25, in accordance with the standard significance thresholds recommended for GSEA.

### 4.7. Total ROS/Superoxide Detection

Following the manufacturer’s protocol, reactive oxygen species (ROS) levels were measured using the ROS-ID Total ROS/Superoxide Detection Kit (ENZ-51010, Madison, NY, USA). CWH43ov and CWH43-KD cells (6.0 × 10^5^) were seeded in six-well plates and incubated overnight. Cells were then treated with specific drugs or vehicle controls. After treatment, cells were harvested and stained with two fluorescent dyes: a green dye to detect total ROS levels and an orange dye to detect superoxide levels in live cells. Additionally, Hoechst-33342 was used to stain the harvested cells, enabling visualization of the total cell population. The NucleoCounter NC-3000 system (ChemoMetec A/S, Allerod, Denmark) measured fluorescence intensity, indicative of ROS and superoxide levels. Results reflect real-time oxidative stress and superoxide levels in CWH43ov and CWH43-KD HCT 116 cells under different treatment conditions.

### 4.8. Lipid ROS Measurement by Flow Cytometry

Lipid ROS levels were measured using C11-BODIPY 581/591 staining followed by flow cytometric analysis. Briefly, DLD-1 and HCT 116 cells were seeded in 6-well plates and allowed to attach overnight. Cells were then treated with erastin (5 μM) in the presence or absence of ferrostatin-1 (0.1 μM) for 6 h. Following treatment, cells were incubated with C11-BODIPY 581/591 (2 μM) at 37 °C for 30 min in the dark. Cells were subsequently washed twice with phosphate-buffered saline (PBS), harvested by trypsinization, collected by centrifugation, and resuspended in PBS for analysis.

Fluorescence signals were acquired using a BD FACSVerse™ flow cytometer (BD Biosciences, San Jose, CA, USA). Oxidized C11-BODIPY fluorescence was detected in the FITC channel, whereas reduced C11-BODIPY fluorescence was detected in the PE channel. Lipid ROS levels were quantified as the ratio of oxidized (FITC) to reduced (PE) fluorescence intensity (FITC/PE ratio). Representative histograms were analyzed using FlowJo software (BD Biosciences). Data were obtained from three independent biological experiments and are presented as mean ± SD. Statistical analyses were performed as described in Section 4.13.

### 4.9. Measurement of Total Glutathione (T-GSH)

Intracellular total glutathione (T-GSH) levels were measured using a Total Glutathione (T-GSH)/Oxidized Glutathione (GSSG) Colorimetric Assay Kit (Cat. No. E-BC-K097-M, Elabscience, Wuhan, China) according to the manufacturer’s instructions. Briefly, cells were harvested and washed with cold PBS. Approximately 1 × 10^6^ cells were homogenized in 200 μL of protein precipitant on ice, followed by centrifugation at 10,000× *g* for 10 min at 4 °C. The supernatant was collected and kept on ice for subsequent analysis. For the T-GSH assay, 10 μL of each sample or standard was added to a 96-well plate, followed by 150 μL of reaction working solution. After incubation at room temperature for 5 min, 50 μL of substrate working solution was added and mixed thoroughly. The reaction was incubated at room temperature for 25 min, and absorbance was measured at 412 nm using a microplate reader. T-GSH concentrations were calculated from a standard curve and normalized to cell number.

### 4.10. Quantitative Real-Time PCR (RT-qPCR) Analysis

Total RNA was extracted from cultured cells using TRIzol reagent (Invitrogen Life Technologies, Carlsbad, CA, USA) according to the manufacturer’s instructions. First-strand cDNA was synthesized from 1 μg of total RNA using a cDNA Synthesis Kit (Invitrogen Life Technologies) in a final reaction volume of 20 μL. Quantitative real-time PCR was performed using SYBR Green Master Mix (Applied Biosystems, Foster City, CA, USA) on an ABI 7500 Fast Real-Time PCR System. The PCR reaction conditions were set as follows: an initial denaturation at 95 °C for 10 min, followed by 40 cycles of 95 °C for 15 s (denaturation) and 60 °C for 60 s (annealing and extension). The primer sequences were as follows: SLC7A11 forward, 5′-TCCTGCTTTGGCTCCATGAACG-3′ and reverse, 5′-AGAGGAGTGTGCTTGCGGACAT-3′; NRF2 forward, 5′-TGCCCACATTCCCAAATCA-3′ and reverse, 5′-TGCGCCAAAAGCTGCAT-3′. Glyceraldehyde-3-phosphate dehydrogenase (GAPDH) was used as the internal control for normalization. Relative expression levels of SLC7A11 and NRF2 were normalized to GAPDH and calculated using the 2^−ΔΔCt^ method. All reactions were performed in triplicate, and each experiment was independently repeated at least three times [60].

### 4.11. Western Blot Analysis

Cells were lysed in radioimmunoprecipitation assay (RIPA) buffer (50 mM Tris-HCl, pH 7.4, 150 mM NaCl, 1% NP-40, 0.5% sodium deoxycholate, 0.1% SDS, and 1 mM EDTA) supplemented with protease and phosphatase inhibitor cocktails (Single-Use Cocktail, Thermo Fisher Scientific, Waltham, MA, USA). Protein concentrations were determined using a bicinchoninic acid (BCA) protein assay kit. Equal amounts of protein (20–40 μg) were separated by SDS-PAGE and transferred onto nitrocellulose membranes using the iBlot™ 3 Dry Blotting System (Invitrogen, Thermo Fisher Scientific, Waltham, MA, USA) according to the manufacturer’s instructions. The membranes were blocked with 5% non-fat milk in PBST for 1 h at room temperature and incubated overnight at 4 °C with primary antibodies. Primary antibodies against CWH43 (rabbit polyclonal; Abcam, Cambridge, UK; Cat# ab185120; 1:1000), GPX4 (rabbit polyclonal; Affinity Biosciences, Cincinnati, OH, USA; Cat# DF6701; 1:1000), SLC7A11 (rabbit monoclonal; Cell Signaling Technology, Danvers, MA, USA; Cat. No. 12691; 1:1000), NRF2 (rabbit polyclonal; iReal Biotechnology, Taipei, Taiwan; Cat# IR136-543; 1:1000), and GAPDH (rabbit polyclonal; iReal Biotechnology, Taipei, Taiwan; Cat# IR3-8; 1:1000) were used. After washing with PBST (phosphate-buffered saline containing 0.1% Tween-20), membranes were incubated with horseradish peroxidase (HRP)-conjugated secondary antibodies (goat anti-rabbit IgG-HRP; Jackson ImmunoResearch, West Grove, PA, USA; Cat# 111-035-003; 1:5000) for 1 h at room temperature. Protein bands were visualized using an enhanced chemiluminescence (ECL) detection system and captured using a ChemiDoc imaging system. GAPDH was used as an internal loading control. Relative protein expression levels were quantified by densitometric analysis using ImageJ software (v1.54, National Institutes of Health, Bethesda, MD, USA).

### 4.12. Mitochondrial Membrane Potential Assay

Mitochondrial membrane potential was assessed using JC-1 (5,5′,6,6′-tetrachloro-1,1′,3,3′-tetraethylbenzimidazolylcarbocyanine iodide) according to the manufacturer’s instructions (MitoProbe™ JC-1 Assay Kit, Thermo Fisher Scientific, Cat. No. M34152). Briefly, DLD-1 and HCT 116 cells with stable CWH43 knockdown or overexpression, together with their respective controls, were seeded in 6-well plates and allowed to attach overnight. Cells were then treated with erastin (5 μM) in the presence or absence of ferrostatin-1 (0.1 μM) for 6 h. Following treatment, cells were incubated with JC-1 working solution at 37 °C for 20 min in the dark. Cells were subsequently washed twice with phosphate-buffered saline, harvested by trypsinization, collected by centrifugation, and resuspended for flow cytometric analysis. Fluorescence signals were acquired using a BD FACSVerse™ flow cytometer (BD Biosciences, San Jose, CA, USA). JC-1 monomers, indicative of low mitochondrial membrane potential, were detected in the FITC (green) channel, whereas JC-1 J-aggregates, indicative of high mitochondrial membrane potential, were detected in the PE (red) channel. Mitochondrial membrane potential was quantified as the ratio of green to red fluorescence intensity, with a higher ratio indicating greater mitochondrial depolarization. For comparison across independent experiments, the green/red fluorescence ratio was normalized to the corresponding untreated control group.

### 4.13. Statistical Analysis

Statistical analyses were performed using GraphPad Prism 9.0 (GraphPad Software, San Diego, CA, USA). Western blot densitometric values obtained from matched independent biological experiments were analyzed using a two-tailed paired Student’s *t*-test. Comparisons between two independent groups were performed using a two-tailed unpaired Student’s *t*-test. Comparisons among three or more groups involving a single experimental factor were performed using one-way analysis of variance (ANOVA), followed by Tukey’s multiple-comparisons test. Experiments involving two experimental factors, such as genetic manipulation and pharmacological treatment, were analyzed using two-way ANOVA, followed by Šídák’s multiple-comparisons test. Unless otherwise indicated, each experiment was independently repeated at least three times. Statistical analyses were performed using values obtained from independent biological experiments rather than technical replicates. Data are presented as mean ± standard deviation (SD), and the exact number of biological replicates is specified in the corresponding figure legends. A two-sided *p* value < 0.05 was considered statistically significant. Statistical significance is indicated as follows: * *p* < 0.05, ** *p* < 0.01, and *** *p* < 0.001.

## 5. Conclusions

In summary, this study identifies CWH43 as a previously underappreciated modulator associated with chemotherapy response, redox adaptation, and ferroptosis-related vulnerability in colorectal cancer. By linking CWH43 expression to stress-adaptive survival programs, our findings provide a mechanistic framework that may inform future studies aimed at overcoming chemotherapy resistance, potentially involving redox-adaptive and ferroptosis-associated pathways.

## Data Availability

The dataset supporting the conclusions of this article is included within the article.

## Supplementary Materials

The following supporting information can be downloaded at: https://www.mdpi.com/article/10.3390/ijms27198830/s1. Figure S1: GSEA demonstrating negative associations between CWH43 expression and multiple hallmark pathways, including apical junction, allograft rejection, interferon alpha response, in-terferon gamma response, epithelial-mesenchymal transition, and coagulation, based on TCGA COADREAD data; Figure S2: CWH43 modulates oxidative stress and superoxide levels in a bidi-rectional manner. CWH43 overexpression decreases (A), while CWH43 knockdown increases (B), oxidative stress and superoxide production. Fluorescence intensity was quantified with the Nu-cleoCounter NC-3000 system and is presented as the mean fluorescence intensity ±SD of the indi-cated number of independent experiments. Symbols denote the values obtained in each individual independent experiment, and lines connect the paired conditions acquired within the same ex-periment. Because each condition and its corresponding control were acquired within the same independent experiment, directional paired one-tailed Student’s *t*-tests were used. * *p* < 0.05, ** *p* < 0.01, *** *p* < 0.001; Figure S3: NRF2 mediates the effects of CWH43 on oxidative stress and super-oxide levels. NRF2 inhibition (A) or activation (B) reverses the effects of CWH43 overexpression and knockdown on oxidative stress and superoxide respectively.
